# Molecular evidence for a diverse green algal community growing in the hair of sloths and a specific association with *Trichophilus welckeri *(Chlorophyta, Ulvophyceae)

**DOI:** 10.1186/1471-2148-10-86

**Published:** 2010-03-30

**Authors:** Milla Suutari, Markus Majaneva, David P Fewer, Bryson Voirin, Annette Aiello, Thomas Friedl, Adriano G Chiarello, Jaanika Blomster

**Affiliations:** 1 Department of Environmental Sciences, P.O.Box 65, FIN-00014, University of Helsinki, Helsinki, Finland; 2 Tvärminne Zoological Station, J.A. Palménin tie 260, FIN-10900 Hanko, Finland; 3 Department of Food and Environmental Sciences, P.O. Box 66, FIN-00014, University of Helsinki, Helsinki, Finland; 4 Max-Planck Institute for Ornithology, Schloß Möggingen, Schloßallee 2, 78315 Radolfzell, Germany; 5 Smithsonian Tropical Research Institute, Apartado 0843-03092 Balboa, Ancon, Rep. Panama; 6 Albrecht-von-Haller-Institut für Pflanzenwissenschaften, Abteilung Experimentelle Phykologie and Sammlung für Algenkulturen, Universität Göttingen, Untere Karspüle 2, 37073 Göttingen, Germany; 7 Catholic University of Minas Gerais, Programa de pós-graduação em Zoologia de Vertebrados, Av. Dom José Gaspar 500, Prédio 41, Belo Horizonte, MG, CEP: 30.535-610, Brazil

## Abstract

**Background:**

Sloths are slow-moving arboreal mammals inhabiting tropical rainforests in Central and South America. The six living species of sloths are occasionally reported to display a greenish discoloration of their pelage. *Trichophilus welckeri*, a green algal species first described more than a century ago, is widely believed to discolor the animals fur and provide the sloth with effective camouflage. However, this phenomenon has not been explored in any detail and there is little evidence to substantiate this widely held opinion.

**Results:**

Here we investigate the genetic diversity of the eukaryotic community present in fur of all six extant species of sloth. Analysis of 71 sloth hair samples yielding 426 partial 18S rRNA gene sequences demonstrates a diverse eukaryotic microbial assemblage. Phylogenetic analysis reveals that sloth fur hosts a number of green algal species and suggests that acquisition of these organisms from the surrounding rainforest plays an important role in the discoloration of sloth fur. However, an alga corresponding to the morphological description of *Trichophilus welckeri *was found to be frequent and abundant on sloth fur. Phylogenetic analysis demonstrated the retention of this alga on the fur of sloths independent of geographic location.

**Conclusions:**

These results demonstrate a unique diverse microbial eukaryotic community in the fur of sloths from Central and South America. Our analysis streghtens the case for symbiosis between sloths and *Trichophilus welckeri*.

## Background

The extant sloths comprise six species of medium-sized slow-moving mammals of Central and South America. They are represented by two distantly related genera *Bradypus*, three-toed sloths, and *Choloepus*, two-toed sloths. The genus *Bradypus *has four extant species: *Bradypus variegatus*, *B. tridactylus*, *B. torquatus *and *B. pygmaeus*. *Bradypus variegatus*, the brown-throated sloth, is the most widespread species and inhabits Central and Southern American rainforests, whereas *B. tridactylus*, the pale-throated sloth, is restricted to northern South America. *Bradypus torquatus*, the maned three-toed sloth, occupies a diminishing area of coastal forests of eastern Brazil, and *B. pygmaeus*, the pygmy three-toed sloth, only recently described, is endemic to the small island, Isla Escudo de Veraguas on the Atlantic coast of Panama [1]. The genus *Choloepus *is represented by two living species: *Choloepus hoffmanni*, the Hoffmann's two-toed sloth, and *C. didactylus*, the southern two-toed sloth. *Choloepus hoffmanni *occurs in Central America, and in South America close to the equator, and *C. didactylus *is restricted to northern South America [2].

Sloth hair is long and coarse and that of two-toed sloths is unique in having a number of deep grooves running the length of each hair, whereas three-toed sloth hair has irregular transverse cracks that increase in number and size with age [3,4]. A wide variety of organisms have been reported to occur in the grooves and cracks of sloth hair, including cyanobacteria and diatoms; and among their fur, moths, beetles, cockroaches and roundworms [5-7]. However, the greenish color of the hair, which is most evident in three-toed sloths, is due to green algae, which in most cases have been identified as *Trichophilus welckeri *[6,8-10]. It is a popular assumption that the association between the three-toed sloth and the alga embedded in its hairs is a symbiotic relationship with the alga obtaining shelter in the cracks of the hair while providing green camouflage for the sloth. It also has been proposed that the alga offers no benefit to the sloth, but is able to survive in the hair because the sloth does not prevent the alga growing [3]. The hair of the three-toed sloth absorbs water like a sponge, perhaps making it an even more ideal habitat for algae, and prompting speculations that the sloth perhaps receives nutrients from the alga via diffusion along the spongy outer portion of the hairs, followed by absorption into the skin [3]. The algae growing on sloth hair may also produce exopolymeric substances that may give the hair a desired texture or allow beneficial bacteria to grow. Karsten et al. [11] found UV-absorbing mycosporine-like amino acid (324 nm-MAA) from a green alga found in sloth hair. Reduced exposure to UV-light could also be seen as beneficial to the sloth over the long run. However, there is no clear evidence supporting any hypothesis at present.

Here we collected 71 sloth hair samples from all six extant sloth species as well as 12 environmental samples from tree trunks to survey the diversity of eukaryotic organisms present there. We found evidence for the acquisition of green algae from the environment as well as retention of green algae in the hair independent of the sloths geographic location.

## Results

**Figure 1:**
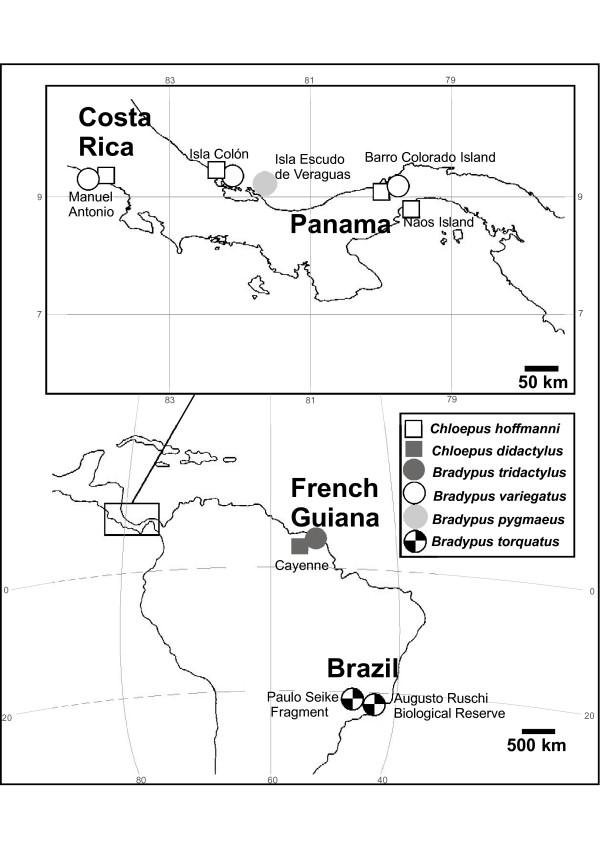
**A map of sampling sites and sloths species sampled in the area**.

**Figure 2:**
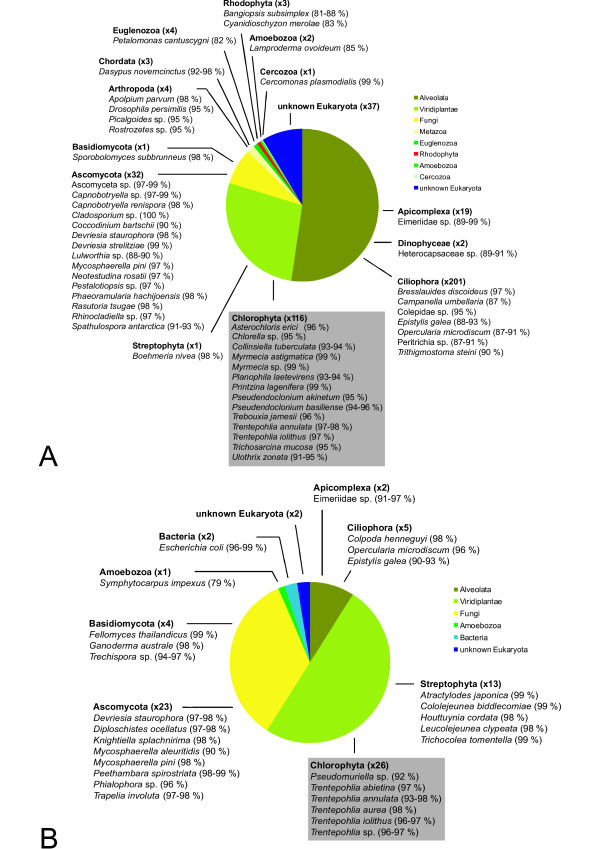
**Diversity of all eukaryotic taxa sequenced from sloth samples**. (A) Diversity of eukaryotic taxa sequenced from all samples (B) Diversity of eukaryotic taxa sequenced from environmental samples collected from Barro Colorado Island, Panama. Taxa names were assigned to the closest known matches in GenBank and it is possible that they represent undescribed or unsequenced species related to the taxa listed in GenBank. Percentage of similarity is given after each taxon name.

Hair samples were examined from 71 sloth individuals, which originated from French Guiana, Panama, Costa Rica and Brazil (Fig. 1). Clone libraries were constructed and 426 eukaryotic 18S rRNA gene sequences were obtained from a total of 72 taxa (Table 1). The number of eukaryotic taxa per sample varied between 12 and 22, except in *C. didactylus *where the only two sequences recovered turned out to represent the same taxon (Table 1). The estimated total number of taxa (S_Chao1 _at the 97% similarity) varied between 21 and 38 being 146 for the total dataset. The Shannon diversity index with 95% confidence intervals was equal in the sloth species containing a minimum of 12 taxa. The Shannon diversity index for the total dataset was 3.1. The coverage, which estimates whether the clone library reflects the actual diversity in the samples, was 89.3% for the complete dataset. In the comparison of different geographical areas the highest number of taxa was found on Isla Escudo de Veraguas, Panama, where the number of taxa was 22 and the estimated number of taxa was 38 (Table 2). The Shannon diversity was lower in Costa Rica (1.4 ± 0.3) and Paulo Seike Fragment (1.5 ± 0.4) than in other areas (ranging from 1.7 ± 0.3 to 2.2 ± 0.5). More than half of the taxa consisted of ciliates, apicomplexans and dinoflagellates (Fig. 2a). One third of the sequences were green algae. Fungi also were present in larger numbers and in addition a few 18S rRNA gene sequences from arthropods, Euglenozoa and red algae, were found. 10% of sequences could not be attributed to a known eukaryote (Fig. 2a).

**Table 1:** Parameters for eukaryotic sequences according to source sloth species.*

Species	Sequences	Number of taxa	Coverage (%)	Estimated n of taxa (S_Chao1_)	Shannon diversity index
*C. hoffmanni*	116	21	89.7	37	2.1 ± 0.2
*C. didactylus*	2	1	-^*a*^	1	0
*B. tridactylus*	33	12	78.8	33	2.2 ± 0.3
*B. variegatus*	63	14	88.9	21	2.0 ± 0.3
*B. pygmaeus*	111	22	89.2	38	2.2 ± 0.2
*B. torquatus*	58	20	81.0	26	2.1 ± 0.4
**Total**	**383**	**72**	**89.3**	**146**	**3.11 ± 0.15**

*The number of taxa, estimated number of taxa and the Shannon diversity index from each sloth species. Shannon index values include the 95% confidence intervals. Coverage estimates if the clone library reflects the actual diversity in the samples. A taxon is defined here as having greater than 97% similarity. ^a^Coverage not calculated for *C. didactylus *due to the low number of sequences.

**Table 2:** Parameters for eukaryotic sequences according to collection site.*

Collection location	Sequences	Number of taxa	Coverage (%)	Estimated n of taxa (S_Chao1_)	Shannon diversity index
Naos Island, Panama	18	10	55.6	38	2.0 ± 0.5
Barro Colorado Island, Panama	37	10	83.8	17	1.7 ± 0.3
Isla Colón, Panama	90	15	93.3	20	2.1 ± 0.2
Isla Escudo de Veraguas,	111	22	89.2	38	2.2 ± 0.2
Panama					
Manuel Antonio, Costa	34	6	94.1	7	1.4 ± 0.3
Rica					
Cayenne, French Guiana	35	12	82.9	27	2.2 ± 0.3
Augusto Ruschi, Brazil	24	12	66.7	19	2.0 ± 0.5
Paulo Seike Fragment,	34	10	85.3	12	1.5 ± 0.4
Brazil					
**Total**	**383**	**72**	**89.3**	**146**	**3.11 ± 0.15**

*The number of taxa (with 97% similarity), estimated number of taxa and the Shannon diversity index from each study area. Shannon index values include the 95% confidence intervals.

**Figure 3:**
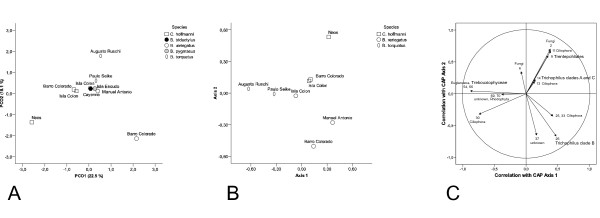
**CAP-analysis of the 97% taxa as variables and collection localities and species as samples**. *C. didactylus *from Cayenne, French Guiana (two sequences) and *C. hoffmanni *from Manuel Antonio, Costa Rica (three sequences) have been excluded from this analysis. (A) The principal coordinate analysis (B) The canonical analysis of *C. hoffmanni*, *B. torquatus *and *B. variegatus*. (C) Correlations of taxa with the two canonical analysis axes from Fig. 3B. Only taxa which had correlation > 0.2 were included. The numbers at the arrowheads denote the 97% taxa listed in Additional file 3.

PCO analysis of all eukaryotic 18S rRNA gene sequences from the sloth hair grouped most samples together, excluding the Naos and Augusto Ruschi samples and the Barro Colorado Island *Bradypus variegatus *-samples (Fig. 3a). The first two PCO axes explained 40.6% of the variability. However, the canonical analysis of the three species of sloths that had been sampled from several locations showed a separation of the different sloth species with squared canonical correlations of δ^2^_1 _= 0.778 and δ^2^_2 _= 0.649 (Fig. 3b). The canonical test statistic (*t*_2_) was significant (*P *= 0.007 with 9999 permutations). The CAP program determined the optimum amount (*m*) of principal coordinates for the canonical analysis to be 3, which achieved maximum proportion of correct allocations (75%) and which explained 65.8% of the variability. Correlations of taxa with both CAP axes are shown graphically in Fig. 3c. We did not include in the figure any taxa with correlations to both axes <0.20 nor ones which occurred only once. The analysis showed that *C. hoffmanni*, *B. torquatus *and *B. variegatus *harbour different taxa in their hair (Fig. 3c). Due to small amount of data from dry season we could not test the separation of samples in relation to dry and wet season. However, Naos Island *C. hoffmanni *and over half of Barro Colorado *B. variegatus *sequences were collected on dry season and the position of those samples in PCO indicates some degree of separation.

Fifty-two of the 71 sloth hair samples studied contained green algae, as observed either directly due to the greenish color of the hair, or by using light microscopy, which in some cases revealed algae even if the color of the hair was dark but not green. Green algal sequences were obtained successfully from 27 of the 71 samples. We were not able to get a PCR product for the remaining 25 samples that contained visible green algae. Green algal 18S rRNA gene sequences were obtained from five extant sloth species. Despite all efforts no green algal 18S rRNA gene sequences were obtained from the two hair samples of *Choloepus didactylus*. Green algae were neither found in microscopic examinations of these two samples. An average of 27% of the sloth samples for which 18S rRNA gene clone libraries were constructed contained green algae (Fig. 2a).

**Figure 4:**
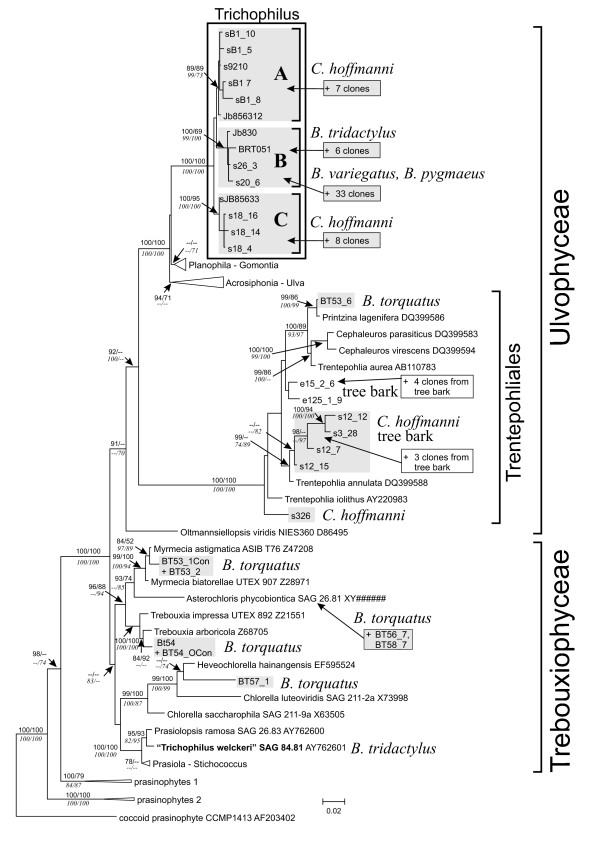
**Phylogenetic tree of the green algae growing on sloth hair**. Maximum likelihood (ML) phylogenetic analysis of 18S rRNA gene clones representing green algae growing on the hair of five species of sloths (highlighted in grey) and bark of tropical trees, their next closest relatives from the classes Ulvophyceae and Trebouxiophyceae and five species of prasinophytes. The phylogeny shows the best ML tree inferred using the programme GARLI v0.96. Numbers at internal branches are bootstrap values (> 65%; from MP and ME analyses above lines, from NJ and GARLI ML below lines) and are given only at those internal nodes which were also resolved by PAUP ML and Bayesian analyses (with posterior probabilities > 0.95). The strain previously assumed to represent the sloths hair alga, *Trichophilus welckeri*, is marked in bold. Sequences except those where accession numbers are given are reported for the first time in this study. Clones whose phylogenetic positions have been inferred by adding their sequences to this ML phylogeny in ARB (see text) but which were not used to construct the phylogeny were simply added to the figure (marked by "+"). Additional rRNA gene clones from sloth hair samples and tree bark that were positioned into clades A-C and the Trentepohliales, respectively are listed in Additional file 4. Some branches were compressed into triangles for clarity of the graphic, the sequences which they contain are listed in Additional file 5.

**Figure 5:**
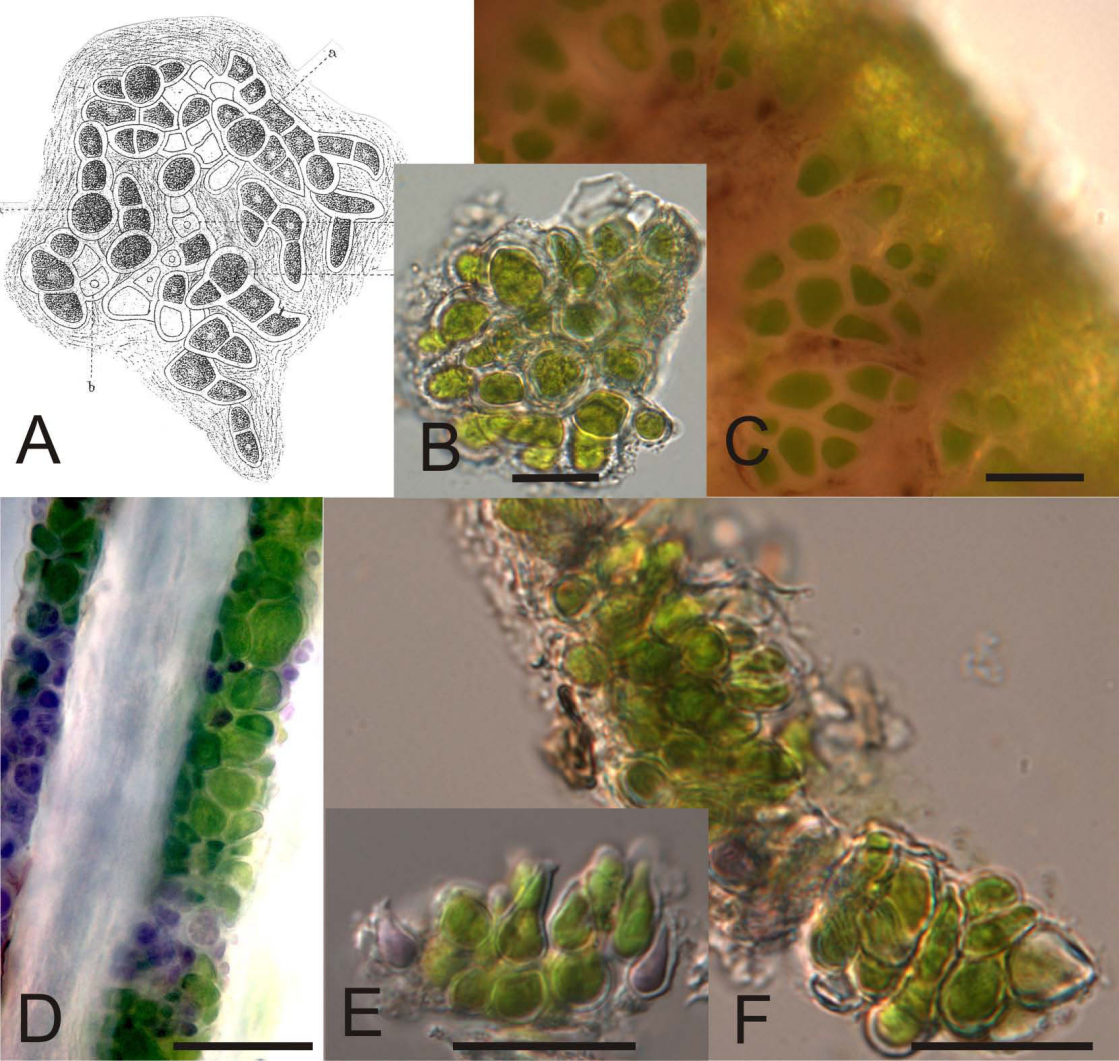
**Morphology of the green alga *Trichophilus welckeri *found in sloth hair**.(A) *Trichophilus welckeri *developing fronds as described in Weber-van Bosse (1887, Fig. 15) [8]. "*a*" refers to sporangia and "*b*" to empty sporangial cells from which spores have been released. (B) *Trichophilus*-like alga from *Bradypus pygmaeus*, sample S26; squash preparation of the algae growing on the hair. (C) Cells of the *Trichophilus*-like alga from *Bradypus pygmaeus*, sample S26. (D)*Choloepus hoffmanni *hair, sample s9 with *Trichophilus*-like alga growing on the hair. (E), (F) Squash preparations of the algae growing on the hair of *Choloepus hoffmanni *sample sB1. Scale bars in all figures 20 μm.

The majority of the green algal sequences obtained from the hair samples formed a clade of their own within the green algal class Ulvophyceae, distinct from other publicly available taxa. This group received high support (100%) for its monophyly in all phylogenetic analyses that were employed (Fig. 4). Based on the morphological characters, small (3-13 μm) thick-walled cells without pyrenoids (Fig. 5), the clade is likely to correspond to the green algal genus *Trichophilus*, which has been described by Weber-van Bosse in 1887 [8,12]. Interestingly, there were no other green algal species found in the same hair samples in which *Trichophilus *was found. The clade was subdivided into three subclades that received moderate to high internal node support. Two clades, A and C in (Fig. 4) were formed by rRNA gene clones originating from *Choloepus hoffmannii *hair samples. Clones in the clade B originated from the hair of *Bradypus*. Within clade B, the sequences from *B. tridactylus *were different from those of other *Bradypus *species; there were four synapomorphic positions at the 5'-end of the 18S rRNA gene that distinguished the *B. tridactylus *hair alga from the corresponding alga of other *Bradypus *species. The bootstrap values for the clade B, originating from hair samples of *Bradypus*, was high (99-100%) in MP, NJ, GarliML analyses and 69% in ME analyses (Fig. 4). Clades A and C, which originated from *Choloepus*, were also supported by high bootstrap support (clade A: 73-99%, clade B: 95-100%, Fig. 4). Separately from the Ulvophycean green algal clade, green algae from sloth hair grouped with several *Trentepohlia*-species, including *Trentepohlia annulata*, as well as with *Prinzina lagenifera*, *Myrmecia *sp., *Asterochloris *sp. and *Chlorella*-like algae. The bootstrap supports for these clades were up to 100%. These samples all came from *Bradypus torquatus *from Brazil and *Choloepus hoffmanni *from Panama. One of the Brazilian samples, BT53, contained two taxa that clearly grouped separately in the phylogenies (*Printzina *and *Myrmecia*; Fig. 4).

We obtained 78 sequences from the 12 environmental samples from Barro Colorado Island. Half of the sequences were green algal or from higher plants. Fungi were also abundant and 10% of sequences were from ciliates and other alveolites (Fig. 2b). All green algal sequences from tree bark were attributable to the genus *Trentepohlia*, a terrestrial green algal genus occurring both in temperate regions as well as in tropics. They formed several independent lineages within the Trentepohliales clade of the 18S rRNA gene phylogeny (Fig. 4).

## Discussion

We found evidence for three different patterns of algae that occur in the hair of five sloth species: 1) The green alga in the fur of the brown-throated sloth, *Bradypus variegatus*, and the pygmy three-toed sloth, *B. pygmaeus*, is a unique species and no other green algal species were found in their fur. Microscopy of the alga on the hair revealed characteristic features to the description of *Trichophilus *(Weber-van Bosse 1887), which has indeed been described from the hair of *Bradypus*. 2) The maned three-toed sloth *Bradypus torquatus *was shown in our study to host a variety of algae belonging to genera known to be terrestrial, e.g. *Trentepholia *and *Myrmecia*. 3) The Hoffmann's two-toed sloth *Choloepus hoffmanni *and the pale-throated sloth *Bradypus tridactylus*, showed both patterns; they hosted terrestrial green algae from their surroundings, as well as the unique genus *Trichophilus*.

The green alga *Trichophilus *that was found in *B. variegatus, B. pygmaeus *and *B. tridactylus *was separated in the phylogenetic trees from the *Trichophilus *occurring in *C. hoffmanni*. The *Bradypus*-clade was supported by high bootstrap values and as *Bradypus variegatus *and *Choloepus hoffmanni *inhabit the same geographic area and habitat [13], the separation of *Trichophilus *inhabiting *Bradypus *suggests co-evolution between *Trichophilus *and *Bradypus *species. This is further supported by the cell sizes of *Trichophilus *in *Bradypus *and *Trichophilus *in *Choloepus *being different.

*Trichophilus welckeri *[8] has been described from sloth hair and generally has been thought to be the only green alga infesting sloth hair [6,9-11]. However, in former phylogenetic studies [10,11]*T. welckeri *(strain SAG 84.81) was shown to group with *Prasiolopsis ramosa*, which is contrary to our study. We suspect earlier studies to be erroneous species identification, made because of its habitat and the morphological investigation of the strain supports this. The strain SAG 84.81 resembles *Prasiolopsis *by forming irregular cushion-like tufts of pseudoparanchymatous filaments and cells with single, stellate, pyrenoid-containing chloroplast typical of the Prasiolales [10] rather than numerous small, discoid chloroplasts without pyrenoids described for *Trichophilus *[12]. Therefore our understanding is that *Trichophilus welckeri *has been found previously in nature [6,8,9,14] but has not yet been isolated into pure culture and sequenced before. It is likely not to occur in any other environment besides sloth hair given that tropical terrestrial green algal species like *T. welckeri *have not been found before. Its restricted occurrence may be explained by it being host-specific and possibly thermophilic.

Among all 71 sloth individuals studied, 73% hosted algae in their fur. It was either observed by the greenish color of the fur or seen under the microscope in closer observation. Seven of the 19 individuals in which we found no evidence of algae were babies still at the age of clinging to their mothers suggesting that at least *Trichophilus *is gained in childhood, most likely from the mother. This observation is supported by an earlier study noting that sloths gain the algae and other parasites by the time they are a few weeks old [5]. Besides *Trichophilus*, other green algae found in sloth hair were terrestrial *Trentepohlia*, *Prinzina*, *Myrmecia *and *Trebouxia *-species as well as *Chlorella*-like algae. Among these, only *Trentepohlia *has been reported previously from sloth hair [6]. It is possible that sloths gain these algae from the environment and host them in their fur. However, they may also represent sloth-inhabiting specialists at a finer evolutionary level, e.g. species level, than *Trichophilus *does. That there were 25 hair samples containing visible green algae but for which we were unable to get PCR-product, suggest that there were either too few algae in the sample or there were PCR inhibitors present in the DNA extracts. This problem could be overcome by culturing the microbial community from fresh hair samples. The diversity of other eukaryotic organisms in the hair was high, as expected, based on previous studies, which have observed a wide range of animals, e.g. moths, beetles, cockroaches, and roundworms [5]. However, a surprising number and diversity of ciliates and fungi were also found in the hair of all sloth species, excluding *C. didactylus*, for which only two sequences were analyzed. All geographical areas studied, except Costa Rica, had similar levels of eukaryote diversity. The similar diversity is likely to be explained by the similar habitat of sloths, regardless of the geographical area they inhabit. However, regardless of the similar diversity, the species composition of eukaryotic organisms varied as was seen in the PCO and CAP-analyses (Fig. 3). The eukaryotes in *B. variegatus, B. torquatus *and *C. hoffmanni *were different (Fig. 3). Partly this separation can be explained by the differing green algae inhabiting the hair as was seen in the phylogenetic tree in which *Trichophilus *in *Bradypus *was separated from *Trichophilus *in *Choloepus *(Fig. 4). Similarly, ciliates were different on the three compared species. This can be due to differing hair structure and possibly chemistry as well as differing ecology of the species. It may also reflect the divergence of the two sloth genera about 20 million years ago [15], which may have led to the separation of the cohabiting eukaryotes as well.

The species composition of Naos Island *C. hoffmanni *was clearly different from other *Choloepus *samples from Panama. This may be due to the environment where the sloths are living and the fact that samples were collected on dry season. Naos Island is a small and dry island on the Pacific coast of Panama, receiving on average 1800 mm rainfall per year [16]. Barro Colorado Island and Isla Colón, are located closer to the Atlantic coast of Panama, and therefore are moister, receiving rainfall of 2600-3300 mm per year [16].

The difference in the species composition in Barro Colorado Island *Choloepus *and *Bradypus *was striking (Fig. 3a) as the samples were collected from the same area, although the majority of *Bradypus *sequences were obtained from sloths on dry season. In contrary, *Choloepus *and *Bradypus *samples from Isla Colón were collected from the same area both on wet season being close to each other in the PCO-analyses (Fig. 3a). This suggests that the alteration of dry and wet season may have an effect on the eukaryotic species composition in the hair of sloths.

Despite the differing species compositions on sloth hair, the results (Fig. 2a) show that besides primary producers (27% algae), there are also heterotrophic organisms (52% ciliates, apicomplexans and dinoflagellates) as well as decomposers (8% fungi) in the fur, suggesting that sloth fur supports a versatile small-scale ecosystem. This was not seen in the environmental samples (Fig. 2b) in which 87% of the organisms are primary producers (algae or plants) or decomposers (fungi).

## Conclusions

This is the first comparative survey of the algae and other organisms found in the fur of all six extant species of sloths, and the first study to use molecular methods to explore evolutionary relationships among the algae. We confirm that a great variety of eukaryotes live in sloth fur in addition to the various green algae. We also show the presence of an alga, attributable to at least three species of the genus *Trichophilus*, that has been found only in sloth hair, and thus apparently is passed directly from the mother to the offspring. That finding adds strong support to the idea that the relationship between sloths and at least one particular alga, *Trichophilus *spp., is a symbiotic one.

## Methods

### Sample collection and morphological studies

We examined 71 hair samples from 22 individuals of *Choloepus hoffmanni*, 2 individuals of *C. didactylus*, 10 individuals of *Bradypus tridactylus*, 17 individuals of *B. variegatus*, 12 individuals of *B. pygmaeus *and 8 individuals of *B. torquatus*. The sampled sloths came from French Guiana, Panama, Costa Rica and Brazil. The sampling was done in dry or wet season and details of collection sites, sloth species and dates are presented in the Additional file 1. A small tuft of sloth hair was sampled from a greenish patch, if the animal was visibly green or a darker patch if no greenish coloring was observed. The hair was removed with scissors and preserved in a plastic vial containing silica gel. Samples were stored in silica gel at ambient temperature until further processing, which usually varied from one to three months. All hair samples were studied with a light microscope. If green algae were visibly present they were photographed for further comparison and preserved samples were kept in silica gel for herbarium material. In addition to the sloth hair samples we collected environmental samples from 12 locations on Barro Colorado Island (Additional file 2). The samples were scraped from the surface of tree trunks, or in one case from the surface of a metal pole, from patches which were visibly green. The greenish material was scraped using a scalpel, stored in a plastic vial containing silica gel and stored as described above.

### DNA extraction, PCR, Cloning and Sequencing

DNA was extracted with phenol-chloroform [17] and further purified using the High Pure PCR Template Preparation Kit (Roche), according to the manufacturers' instructions. A 1.5 kb fragment of the 18S rRNA was amplified using the universal primers UNI7F and UNI1534R [18]. However, in some hair samples we were only able to get sequences from other eukaryotes but not from green algae, even though they were clearly visible under the microscope. Therefore, additional primers 501F (5' GGGTCTGGTTTTGAAATGAGG 3') and 1700R (5' CCGAAGTCTTCACCAGCACATC 3') were used to amplify a 1.2 kb fragment of the 18S gene from the green algae. Alternatively a combination of universal and green algal specific primers was used to amplify the green algae present in the samples. A combination of primers 107F (5' CGAATGGCTCATTAAAT 3') and UNI1534R were used to amplify the green algae in the sample BT54_10, which was collected in Brazil.

The PCR amplification was done using Taq DNA polymerase (ABgene) in 25 μl reactions. 0.6 μl Bovine Serum Albumin (BSA, Fermentas, 1 mg/ml) was added into the 25 μl PCR reaction to prevent potential inhibitors in the total DNA carried from hair samples to the PCR reactions. The fragments were amplified in a PCR cycle of initial denaturing of 3 minutes at 94°C, followed by 30 cycles of 1 minute at 94°C, 1 minute at 50°C, 2 minutes at 72°C and a final extension of 5 minutes at 72°C. Products were run on 1% agarose gel and the fragments of desired length were cloned using pGEM^®^-T Easy Vector System II -cloning kit (A1380, Promega) according to the manufacturers' instructions. Positive colonies were picked with a toothpick, dipped into PCR reaction mixture with Taq DNA polymerase (ABgene) and PCR amplified using vector primers T7 (5' TAATACGACTCACTATAGGG 3') and SP6 (5' ATTTAGGTGACACTATAGAA 3').

The PCR products from the clone libraries were purified using Illustra GFX™ PCR DNA and Gel Band Purification Kit (GE Healthcare) or MultiScreen^®^PCR μ96 (Millipore). They were sequenced using primers SP6 and T7 or primers used for PCR amplification (UNI7F, UNI1534R, 501F or 1700R) as well as with internal universal primers 384F (5' ACCACATCCAAGGAWGGCA 3') or 18S384F (5' GGCKACCACAUCCAAGGAWGGCA 3') designed for this study. The samples were loaded on an automated sequencer 3730×l (Applied Biosystems) or ABI3130XL Genetic Analyzer (Applied Biosystems).

### Sequence alignment and phylogenetic analyses

The 18S rRNA gene sequences obtained in this work were manually corrected using Chromas 2.31 (Technelysium, Pty Ltd.) sequence analysis software. Vector sequence was removed and all sequences shorter than 650 bp were excluded from further analysis. The dataset of 426 sequences (hair samples) and 78 sequences (environmental samples) were analyzed using the BLAST network service [19] against nr database at NCBI and results were parsed for taxonomic information.

In order to obtain a reliable alignment for further analyses, 43 sequences of sloth hair that began at the 3' end of the 18S gene were removed. For the remaining 383 sequences operational taxonomic units (taxa) were defined at 97% similarity (Additional file 3) from the MAFFT vs. 6 [20] aligned (1.53 gap opening penalty, 0.123 gap extension penalty) and Phylip version 3.6 [21] -Kimura 2-parameter corrected distance matrix using the furthest neighbor command of the DOTUR program [22]. Coverage [23] was calculated for the whole dataset by the following equation: C = (1 - n/N)*100%, where *C *is the coverage percentage, *n *is the number of taxa (97% similarity) appearing only once, and *N *is the number of all sequences in the library. The estimated number of taxa (S_Chao1_) and Shannon diversity index with 95% confidence intervals were calculated using DOTUR. Principal Coordinate Analysis followed by canonical analysis on PCO axes and calculation of canonical test statistic (*t*_2_) was performed with the program CAP [24,25] using chi-square distance and visualized with SPSS 15. The chi-square distance was chosen since it standardizes differences in scale and emphasizes changes in composition rather than changes in abundance. This was chosen because the abundance of sequences was derived from PCR amplification which is not a real abundance, but a compositional view. The program determined the appropriate number of dimensions (*m*) included in the canonical analysis and identified the taxa that were responsible for multivariate patterns. The number of each 97% taxa was treated as one variable and a sloth species collected from one locality was treated as a sample.

The newly determined green algal 18S rRNA gene sequences were compared to a broad selection of corresponding sequences from members of the Chlorophyta. The closest matching full 18S rRNA gene sequence for each algal clone, as found from BLAST [19] searches, was also included. The selection of sequences was based on a phylogenetic tree comprising an expanded sample of more than 1500 rRNA gene sequences from the green algae which is available in the 18S rRNA gene sequence database maintained in the ARB program [26]. This database was updated with all currently available 18S rRNA gene sequences from the Chlorophyta. Newly determined almost full sequences (>1600 bp long) as well as partial sequences from clone libraries were added to the database using the parsimony interactive tool in ARB. The alignment was refined by comparing the sequences with their next relatives from the ARB database based on their pairing across a helix using secondary structure models as implemented in ARB. This program generates a MP tree from all sequences and all positions in the database as its reference tree, using a filter based on 50% base frequency across all species. A subset of these sequences comprising a total of 63 representatives of the green algal classes Ulvophyceae, Trebouxiophyceae and five prasinophytes (with prasinophyte sequence AF203402 as outgroup) was then downloaded from the ARB database for further analyses using the 50% base frequency filter. The final alignment of 68 taxa (23 algal sequences from sloth hair, 2 from tree bark) was 1690 nucleotides in length. Of the aligned sites, 419 were parsimony informative and an additional 185 were variable but not informative. An optimized maximum likelihood tree (see below) was then uploaded into ARB. To infer the phylogenetic positions of additional rDNA clones (almost full and partial sequences) which were not used for phylogeny construction were added to this tree using the parsimony interactive tool.

Maximum likelihood phylogenies were calculated using PAUP* version 4.0b10 [27] and GARLI v0.96 http://www.nescent.org/wg_garli[28] (Additional file 4 and Additional file 5). ModelTest 3.7 [29] used in conjunction with PAUP 4b10 determined that the TrN+I+G model [30] provided the best fit to the data according the AIC criterion with estimations of nucleotide frequencies (A = 0.254, C = 0.217, G = 0.281, T = 0.248), a rate matrix with six different substitution types, assuming a heterogeneous rate of substitutions with a gamma distribution of variable sites (number of rate categories = 4, shape parameter = 0.60) and a proportion of invariable sites (pinvar) of 0.46. The ML tree obtained in PAUP was used as input for three additional rounds of ML analyses to search for trees with smaller -ln likelihoods, but trees with better likelihood scores were not obtained. Bootstrap resampling was performed on the ML tree obtained in GARLI with 1000 replications. Bayesian phylogenetic analysis was performed with MrBayes version 3.1.2 [31,32] using the GTR+I+G model (rate matrix with six different substitution types, number of rate categories = 4, and with the nucleotide frequencies, shape parameter α, and pinvar estimated from the data), four Markov chains and 2,000,000 generations sampling every 100 generations with the first 25% of the sampled trees discarded, leaving 15,000 trees. Posterior probabilities were then calculated from two independent runs using the 50% majority rule consensus of the kept trees. Minimum Evolution [33], neighbor-joining [34] distance and Maximum Parsimony (MP) approaches were done in PAUP 4b10. ME distance trees were constructed with DNA distances set to maximum likelihood and a heuristic search procedure with 10 random input orders and TBR were employed to find the best tree. Best scoring trees were held at each step. NJ phylogenies were constructed in connection with the "HKY85 model" [35]. In MP analyses, the sites were weighted (RI over an interval of 1-1000). The heuristic search for the best tree was the same as in ME analyses. Bootstrap resampling was performed on NJ trees with 2000, for ME and MP with 1000 replications.

## Authors' contributions

MS collected samples, carried out the molecular systematic work as well as drafted the manuscript. MM and TF performed the statistical and molecular phylogenetic analyses. DPF and JB conceived and designed the study. In addition, JB carried out molecular systematic work and coordinated the study. BV, AA and AGC participated in sample collection and the design of the study. In addition all authors have contributed on the manuscript drafting. All authors read and approved the final manuscript.

## Supplementary Material

Additional file 1 **Sloth sample collection details**. Collection details of the sloth samples which sequence data was used in the study and the number of clones sequenced from each sample. Click here for file

Additional file 2 **Environmental sample collection details**. Collection details of the environmental samples which sequence data was used in the study and the number of clones sequenced from each sample. Click here for file

Additional file 3 **Sequences belonging to a certain taxon with 97% similarity**. Sequences belonging to a certain taxon with 97% similarity. The number in the first column refers to numbers at the arrowheads in Fig. 3c. Click here for file

Additional file 4 **Ulvophycean green algae in **Fig. 4. Additional clones originated from sloth hairs and tree bark which were positioned within the Ulvophyceae in the phylogenetic analyses of Fig. 4. Click here for file

Additional file 5 **Sequences of the clades that were shown compressed in **Fig. 4. Sequences of the clades which were shown compressed in Fig. 4. Click here for file
