# Supplementary material for: Molecular evidence for a diverse green algal community growing in the hair of sloths and a specific association with Trichophilus welckeri (Chlorophyta, Ulvophyceae)
Source: BMC Evol Biol. 2010 Mar 30;10:86. doi: 10.1186/1471-2148-10-86 (PMC2858742; doi:10.1186/1471-2148-10-86)
Supplement: Additional file 1 — Sloth sample collection details. Collection details of the sloth samples which sequence data was used in the study and the number of clones sequenced from each sample. [file 1471-2148-10-86-S1.PDF]

Additional file 1 - Collection details of the sloth samples which sequence data was used in the study and the number of clones sequenced from each sample.

Sample number	Sloth species	Sloth details	Collection place	Collection date	Algal observations	Clones sequen-ced
A101	*Choloepus hoffmanni*	roadkill	Panamerican Highway between Arraiján and Panama City	19.6.2001	-	-
S2	*Choloepus hoffmanni*		Naos Island, Panama	7.1.2006	-	8
S3	*Choloepus hoffmanni*		Naos Island, Panama	7.1.2006	algae visible in the microscope	3
s4M	*Choloepus hoffmanni*		Naos Island, Panama	7.1.2006	algae visible in the microscope	1
S6	*Choloepus hoffmanni*		Naos Island, Panama	7.1.2006	-	4
s6.1	*Choloepus hoffmanni*	baby of s6	Naos Island, Panama	7.1.2006	-	-
S7	*Choloepus hoffmanni*		Naos Island, Panama	7.1.2006	-	2
S8	*Choloepus hoffmanni*	5,8 kg	Barro Colorado Island, Panama	8.1.2006	algae visible in the microscope	9
S9	*Choloepus hoffmanni*	5,2 kg	Barro Colorado Island, Panama	13.1.2006	algae visible in the microscope	3

s11	*Choloepus hoffmanni*	*Terminalia catappa*, Indian almond tree	Isla Colón, Bocas del Toro, Panama	23.6.2006	hair visibly green	3
s12b	*Choloepus hoffmanni*	*Terminalia catappa*, Indian almond tree	Isla Colón, Bocas del Toro, Panama	24.6.2006	hair visibly green	10
s15	*Choloepus hoffmanni*		Isla Colón, Bocas del Toro, Panama	28.6.2006	hair visibly green	4
s16	*Choloepus hoffmanni*	*Terminalia catappa*, Indian almond tree on the shore	Isla Colón, Bocas del Toro, Panama	30.6.2006	hair visibly green	4
s18	*Choloepus hoffmanni*	*Terminalia catappa*, Indian almond tree	Isla Colón, Bocas del Toro, Panama	1.7.2006	hair visibly green	18
s32	*Choloepus hoffmanni*	*Terminalia catappa*, Indian almond tree	Isla Colón, Bocas del Toro, Panama	5.7.2006	hair visibly green	12
s33	*Choloepus hoffmanni*	small individual, *Terminalia catappa*, Indian almond tree	Isla Colón, Bocas del Toro, Panama	5.7.2006	hair visibly green	7

s34	*Choloepus hoffmanni*	*Terminalia catappa*, Indian almond tree	Isla Colón, Bocas del Toro, Panama	5.7.2006	hair visibly green	12
sB1	*Choloepus hoffmanni*		Barro Colorado Island, Panama	7.8.2006	hair visibly green	16
sB3	*Choloepus hoffmanni*		Barro Colorado Island, Panama	1.8.2006	-	-
sJB856	*Choloepus hoffmanni*	old male	Manuel Antonio, Costa Rica	Mar. 2006	hair visibly green	5
850002	*Choloepus hoffmanni*	zoo	Korkeasaari zoo, Helsinki, Finland		-	-
COS1	*Choloepus hoffmanni*	baby	Costa Rica	13.4.2005	-	-
CHD 108	*Choloepus didactylus*	zoo	Cayenne, French Guiana	25.1.2003	-	1
CHD 007	*Choloepus didactylus*		Cayenne, French Guiana	20.02.2003	-	2
BRT 047	*Bradypus tridactylus*	female subadult	French Guiana	14.11.2002	Algae visible in the microscope	-
BRT 048	*Bradypus tridactylus*	female adult	French Guiana	26.10.2002	-	2
BRT 049	*Bradypus tridactylus*	male adult, wounded	French Guiana	11.11.2002	-	4
BRT 050	*Bradypus tridactylus*	1 month old baby	French Guiana	14.11.2002	-	-

BRT 051	*Bradypus tridactylus*	female subadult, forest site	French Guiana	06.1.2003	hair dark, algae visible in the microscope	1
BRT 052	*Bradypus tridactylus*	female adult, forest site	French Guiana	08.1.2003	-	1
BRT 054	*Bradypus tridactylus*		French Guiana	17.2.2003	-	-
BRT 055	*Bradypus tridactylus*	male	French Guiana	14.2.2003	algae visible in the microscope	47
BRT 056	*Bradypus tridactylus*		French Guiana	24.2.2003	-	-
BRT 057	*Bradypus tridactylus*		French Guiana	02.3.2003	-	1
sTRIW	*Bradypus tridactylus*		Brazil, Amazonia	1977	algae visible in the microscope	-
JB812	*Bradypus variegatus*	male adult	Costa Rica	Sept. 2005	hair visibly green	42
JB830	*Bradypus variegatus*	electrocuted sloth	Costa Rica	Nov.2005	hair visibly green	1
s5	*Bradypus variegatus*		Naos Island, Panama	7.1.2006	algae visible in the microscope	-
s10	*Bradypus variegatus*	3,1 kg	Barro Colorado Island, Panama	14.1.2006	algae visible in the microscope	4

s13K	*Bradypus variegatus*		Isla Colón, Bocas del Toro, Panama	25.6.2006	algae visible in the microscope	3
s14	*Bradypus variegatus*	female, on the ground	Isla Colón, Bocas del Toro, Panama	27.6.2006	hair visibly green	3
s14.1	*Bradypus variegatus*	baby of s14	Isla Colón, Bocas del Toro, Panama	27.6.2006	-	-
s17	*Bradypus variegatus*	female, *Cecropia*-tree	Isla Colón, Bocas del Toro, Panama	1.7.2006	hair visibly green	2
s17.1	*Bradypus variegatus*	baby of s17	Isla Colón, Bocas del Toro, Panama	1.7.2006	algae visible in the microscope	-
s30	*Bradypus variegatus*	male, mangrove tree	Isla Colón, Bocas del Toro, Panama	4.7.2006	hair visibly green	11
s31	*Bradypus variegatus*	female adult, mangrove tree	Isla Colón, Bocas del Toro, Panama	4.7.2006	hair visibly green	1
s31.1	*Bradypus variegatus*	baby of s31, mangrove tree	Isla Colón, Bocas del Toro, Panama	4.7.2006	-	-
sB2	*Bradypus variegatus*	female	Barro Colorado Island, Panama	31.7.2006	algae visible in the microscope	5
sB5	*Bradypus variegatus*		Barro Colorado Island, Panama	10.3.2006	-	-
sB6	*Bradypus variegatus*	sick female	Barro Colorado Island, Panama	7.3.2006	hair visibly green	4

sB6.1	*Bradypus variegatus*	sick baby of sB6	Barro Colorado Island, Panama	7.3.2006	algae visible in the microscope	-
s19	*Bradypus pygmaeus*	small individual, mangrove tree	Isla Escudo de Veraguas, Panama	3.7.2006	hair visibly green	9
s20	*Bradypus pygmaeus*	near to s19, mangrove tree	Isla Escudo de Veraguas, Panama	3.7.2006	hair visibly green	14
s21	*Bradypus pygmaeus*	female adult, mangrove tree	Isla Escudo de Veraguas, Panama	3.7.2006	hair visibly green	8
s21P	*Bradypus pygmaeus*	baby of s21, mangrove tree	Isla Escudo de Veraguas, Panama	3.7.2006	-	7
s22	*Bradypus pygmaeus*	female, mangrove tree	Isla Escudo de Veraguas, Panama	3.7.2006	hair visibly green	6
s23n	*Bradypus pygmaeus*	female, mangrove tree	Isla Escudo de Veraguas, Panama	3.7.2006	hair visibly green	11
s24	*Bradypus pygmaeus*	male, mangrove tree	Isla Escudo de Veraguas, Panama	3.7.2006	hair visibly green	6
s25	*Bradypus pygmaeus*	female, mangrove tree	Isla Escudo de Veraguas, Panama	3.7.2006	hair visibly green	11

s26	*Bradypus pygmaeus*	female, mangrove tree	Isla Escudo de Veraguas, Panama	3.7.2006	hair visibly green	13
s27	*Bradypus pygmaeus*	small individual, mangrove tree	Isla Escudo de Veraguas, Panama	3.7.2006	hair visibly green	1
s28S	*Bradypus pygmaeus*	small individual, mangrove tree	Isla Escudo de Veraguas, Panama	3.7.2006	algae visible in the microscope	15
s29	*Bradypus pygmaeus*	male, mangrove tree	Isla Escudo de Veraguas, Panama	3.7.2006	hair visibly green	10
BT 53	*Bradypus torquatus*	adult male 6,7kg	Augusto Ruschi Biological Reserve, Santa Teresa, Espirito Santo, Brazil	29.1.2006	algae visible in the microscope	6
BT 54	*Bradypus torquatus*	adult female 9,2kg	Augusto Ruschi Biological Reserve, Santa Teresa, Espirito Santo, Brazil	30.1.2006	algae visible in the microscope	7
BT 56	*Bradypus torquatus*	adult male 5,7kg	Augusto Ruschi Biological Reserve, Santa Teresa, Espirito Santo, Brazil	30.3.2006	algae visible in the microscope	12

BT 57	*Bradypus torquatus*	adult female 9,5kg	Paulo Seike Fragment, Santa Maria de Jetibá, Espirito Santo, Brazil	31.3.2006	algae visible in the microscope	10
BT 58	*Bradypus torquatus*	adult female 7,1kg	Paulo Seike Fragment, Santa Maria de Jetibá, Espirito Santo, Brazil	31.3.2006	algae visible in the microscope	6
BT 59	*Bradypus torquatus*	infant 0,5kg	Paulo Seike Fragment, Santa Maria de Jetibá, Espirito Santo, Brazil	26.4.2006	-	-
BT 60	*Bradypus torquatus*	adult female 7,3kg	Paulo Seike Fragment, Santa Maria de Jetibá, Espirito Santo, Brazil	26.4.2006	algae visible in the microscope	7
BT 61	*Bradypus torquatus*	adult male 7,6kg	Paulo Seike Fragment, Santa Maria de Jetibá, Espirito Santo, Brazil	26.4.2006	algae visible in the microscope	11
