# Supplementary material for: Molecular evidence for a diverse green algal community growing in the hair of sloths and a specific association with Trichophilus welckeri (Chlorophyta, Ulvophyceae)
Source: BMC Evol Biol. 2010 Mar 30;10:86. doi: 10.1186/1471-2148-10-86 (PMC2858742; doi:10.1186/1471-2148-10-86)
Supplement: Additional file 2 — Environmental sample collection details. Collection details of the environmental samples which sequence data was used in the study and the number of clones sequenced from each sample. [file 1471-2148-10-86-S2.PDF]

Additional file 2 - Collection details of the environmental samples which sequence data was used in the study and the number of clones sequenced from each sample.

Sample number	Sample details	Collection place	Collection date	Clones sequenced
e1	tree bark, 1 m above the ground	Barro Colorado Island, Panama	1.1.2006	7
e4	tree bark, 1 m above the ground	Barro Colorado Island, Panama	1.1.2006	5
e9	tree bark, 1.5 m above the ground	Barro Colorado Island, Panama	1.1.2006	9
e15	fallen tree trunk	Barro Colorado Island, Panama	1.1.2006	10
e17	tree bark, 1 m above the ground	Barro Colorado Island, Panama	1.1.2006	3
e58	tree bark	Barro Colorado Island, Panama	8.1.2006	8
e59	tree bark	Barro Colorado Island, Panama	8.1.2006	1
e86	metal pole	Barro Colorado Island, Panama	12.1.2006	3
e125	exposed *Cecropia* roots	Barro Colorado Island, Panama	16.1.2006	11
e132	tree bark	Barro Colorado Island, Panama	13.6.2006	1
e136	tree bark	Barro Colorado Island, Panama	13.6.2006	5
e138	tree bark	Barro Colorado Island, Panama	17.6.2006	15
