# Supplementary material for: Molecular evidence for a diverse green algal community growing in the hair of sloths and a specific association with Trichophilus welckeri (Chlorophyta, Ulvophyceae)
Source: BMC Evol Biol. 2010 Mar 30;10:86. doi: 10.1186/1471-2148-10-86 (PMC2858742; doi:10.1186/1471-2148-10-86)
Supplement: Additional file 3 — Sequences belonging to a certain taxon with 97% similarity. Sequences belonging to a certain taxon with 97% similarity. The number in the first column refers to numbers at the arrowheads in Fig. 3c. [file 1471-2148-10-86-S3.PDF]

Additional file 3 - Sequences belonging to a certain taxon with 97% similarity. The number in the first column refers to numbers at the arrowheads in Fig. 3c.

1	s2-1
2	s2-2,s2-8,s2-5,CHD007-1,CHD007-2,BRT57-1
3	s2-3
4	s2-4
5	s2-6
6	s2-7,BT60-6,s12-4,BT60-8,s13k-11,s13k-4,s29-7
7	s3-26
8	s3-27
9	s3-28,s12-12,s12-14,s12-15,s12-7,s12-6
10	s4-311
11	s6-1,s6-2,s6-3,s6-4,s7-1,s7-2,s8-1,s8-3,s12-3,s12-10,s16-20,s33-4,s34-10,s34-12,s34-5,s34-3,s34-11,sB1-17,s8-6,s16-6,s11-2,s8-8,s16-8,s8-7,s8-4,s18-1,s11-14,s15-4,s16-1,s18-9,s34-6,s32-3,s34-13,s33-5,s15-14,s34-16,s18-19,sB1-1,sB1-14,sB1-3,s11-13,s12-1,s30-17,s30-5,s30-6
12	s8-2
13	s8-5,s12-13,s15-8,s18-2,s32-11,s32-13,s18-17,s18-18,s32-10,s32-4,s18-5
14	s9-210,sB1-20,sB1-4,sB1-6,sB1-15,sB1-2,sB1-10,sB1-7,sB1-9,sB1-5,sB1-18,sJB856-312,sB1-8,s18-12,s18-4,s18-8,s32-12,s32-7,s18-16,s33-7,s18-14,s32-6,s33-14,sJB856-313,sJB856-33
15	sB1-13,s17-18,s23n-14,s26-4,s26-2,s18-11,BRT-055-4,s23n-13,s19-11,s23n-1,s23n-2,s23n-3,s23n-6,s23n-9,s25-5,s21-15,s23n-7,s21-18,s20-14,s20-9
16	s15-6

17	s18-10
18	s18-20,s32-1,s32-14,s32-15,s32-5,s33-6,s34-7,s18-3,s18-6,s18-7,s34-15,812-24,s17-5,s28s-2,s27-4,s21P-1,s28s-8,s28s-11,s28s-20,s28s-24,s20-16,s28s-16
19	s33-2,s34-4
20	s33-3,BRT049-18F
21	s34-8,s19-5,s23n-15,s26-12
22	BRT048-GRC
23	BRT049-18G
24	BRT049-18R
25	BRT049-18R,BRT-055-20,812-33,812-42,812-38,812-46,812-52
26	BRT051,BRT-055-23,BRT-055-36,BRT-055-44,BRT-055-48,BRT-055-1,BRT-055-10,BRT-055-46,812-15c,812-2,JB830,812-6c,812-8c,812-4c,sB6-2,sB6-7,s30-20,s30-15,s30-2,s21-1ok,s20-6,sB6-5,s23n-16,s25-10,s25-14K2,s24-3,s25-15,s25-6,s25-8,s26-5,s20-10,s26-13,s26-141,s25-2,s26-3,s19-7,s25-11,s25-3,812-23,812-3,812-30c,812-1,812-20,812-39,812-40,812-43,812-53,812-16
27	BRT-055-11,BRT-055-26,BRT-055-39,BRT-055-42
28	BRT-055-13,BRT-055-7,BRT-055-24,BRT-055-47,BRT-055-41
29	BRT-055-14,BRT-055-16,BRT-055-45,BRT-055-9
30	BRT-055-28,812-27,s14-15,s30-10,s30-11,s30-12,s20-12,s20-4,s20-5,s20-8,s21-5,s21P-2,s22-2,s22-28,s24-1,s24-8,s24-9,s26-1,s26-15,s28s-13,s28s-23,s28s-5,s28s-9,s29-10,s29-11,s29-2,s29-4,s29-6,s29-8,s29-9,BT56-0,BT56-4,BT56-8,BT56-9,BT57-0,BT57-8,BT57-9,BT58-3,BT58-5,BT60-2,BT60-9,BT61-0,BT61-1,BT61-5,BT61-6,BT61-7,BT61-9,s31-15,s30-14,s26-6,s26-9,s22-18,s24-7,s24-10,s28s-3,BT61-10,s29-17,BT56-2,BT56-3,BT54-8,BT57-4,s20-0,s20-3,BT61-4,BT58-4,BT58-2,s28s-19,s28s-22,BT58-10,sB2-8,s22-20,s30-

	4,s20-1,s29-14,BT60-10,s14-9,BT56-1,BT56-6,s20-2,BT56-11,s21-25,sB2-14,sB2-5,sB2-6
31	BRT-055-3,BRT-055-37,BRT-055-6
32	812-18
33	812-21,812-41,812-44,812-25,812-31,812-50,812-32
34	s10-1,s10-2
35	s10-3
36	sB2-16
37	sB6-1,s22-25,s19-3,s19-4
38	s13k-8
39	s14-13
40	s19-13
41	s19-2
42	s19-6
43	s19-8,s23n-5
44	s20-15
45	s21-10Con
46	s21-11
47	s21-26
48	s21P-11,s21P-4,s21P-8,s25-1
49	s21P-12
50	s21P-14
51	s22-17
52	s25-17,s26-10
53	s26-8

54	s28s-12,BT54-10,BT60-4
55	s28s-18
56	BT53-1Con,BT53-2
57	BT53-5
58	BT53-6,BT53-8
59	BT53-7
60	BT54-1
61	BT54-4
62	BT54-6
63	BT54-7
64	BT56-10
65	BT56-5
66	BT56-7,BT58-7
67	BT57-1
68	BT57-10,BT57-6
69	BT57-18,BT61-13,BT61-3
70	BT57-2,BT60-3
71	BT57-3
72	BT61-2
