# Supplementary material for: Molecular evidence for a diverse green algal community growing in the hair of sloths and a specific association with Trichophilus welckeri (Chlorophyta, Ulvophyceae)
Source: BMC Evol Biol. 2010 Mar 30;10:86. doi: 10.1186/1471-2148-10-86 (PMC2858742; doi:10.1186/1471-2148-10-86)
Supplement: Additional file 4 — Ulvophycean green algae in Fig. 4. Additional clones originated from sloth hairs and tree bark which were positioned within the Ulvophyceae in the phylogenetic analyses of Fig. 4. [file 1471-2148-10-86-S4.PDF]

Additional file 4 - Additional clones originated from sloth hairs and tree bark that were positioned within the Ulvophyceae in the phylogenetic analyses of Fig. 4.

---

clade A	from *Choloepus hoffmannii*: sB1_2, sB1_4, sB1_6, sB1_9, sB1_15 sB1_18, sB1_20.
clade B	from *Bradypus variegatus* or *B. pygmaeus*: 812_1, 812_2, 812_3, 812_6c, 812_8c, 812_16, 812_20, 812_23, 812_30c, 812_39, 812_40, 812_43, 812_53, s19_7, s20_10, s21_1ok, s23n, s24_3, s25_10, s25_11, s25_14K2, s25_2, s25_6, s25_15, s26_13, s26_141, s26_5, s30_15, s30_2, s30_20, sB6_2, sB6_5, and sB6_7; from *Bradypus tridactylus*: BRT_05542, BRT0551, BRT05511, BRT05526, BRT05546, BRT055yf.
clade C	from *Choloepus hoffmannii*: sB1_2, sB1_4, sB1_6, sB1_9, sB1_15, sB1_18, sB1_20.
tree bark	unidentified Trentepohliales: 15_2_7, 15_2_15, 125_1_14, 132_2; *Trentepohlia annulata*-related: 138_6, 138_12, 15_2_10.
