# Supplementary material for: Molecular evidence for a diverse green algal community growing in the hair of sloths and a specific association with Trichophilus welckeri (Chlorophyta, Ulvophyceae)
Source: BMC Evol Biol. 2010 Mar 30;10:86. doi: 10.1186/1471-2148-10-86 (PMC2858742; doi:10.1186/1471-2148-10-86)
Supplement: Additional file 5 — Sequences of the clades that were shown compressed in Fig. 4. Sequences of the clades which were shown compressed in Fig. 4. [file 1471-2148-10-86-S5.PDF]

Additional file 5 - Sequences of the clades that were shown compressed in Fig. 4.

clade *Planophila - Gomontia*	*Gloeotilopsis paucicellularis* Z47997, *Gomontia polyrhiza* AY278216, *Hazenia mirabilis* AF387156, *Planophila laetevirens* AJ416102, *Pseudendocloniopsis botryoides* AJ416103, *Pseudendoclonium akinetum* DQ011230, *Trichosarcina mucosa* AM109906, *Ulothrix zonata* Z47999
clade *Acrosiphonia - Ulva*	*Acrosiphonia arcta* AY303600, *Ulva intestinalis* AJ000040, *Ulvaria obscura* AY303590, *Blidingia minima* var. *minima* AF499659, *Bolbocoleon piliferum* AY303597, *Desmochloris halophila* AB049416, *Pseudendoclonium basiliense* Z47996, *Pseudoneochloris marina* U41102, *Urospora penicilliformis* AB049417
clade *Prasiola - Stichococcus*	*Desmococcus olivaceus* EU434017, *Prasiola crispa* AJ416106, *Stichococcus bacillaris* EU434029, *Stichococcus bacillaris* AJ416107
clade prasinophytes 1	*Nephroselmis olivacea* X74754, *Nephroselmis marina* X75565
clade prasinophytes 2	*Mantoniella squamata* X73999, *Ostreococcus tauri* Y15814
